# Metal artifact reduction and tumor detection using photon-counting multi-energy computed tomography

**DOI:** 10.1371/journal.pone.0247355

**Published:** 2021-03-05

**Authors:** Chang-Lae Lee, Junyoung Park, Sangnam Nam, Jiyoung Choi, Yuna Choi, Sangmin Lee, Kyoung-Yong Lee, Minkook Cho

**Affiliations:** CT R&D Group, Health & Medical Equipment Business, Samsung Electronics Co., Ltd., Suwon, Republic of Korea; Humanitas Clinical and Research Center - IRRCS, ITALY

## Abstract

Metal artifacts are considered a major challenge in computed tomography (CT) as these adversely affect the diagnosis and treatment of patients. Several approaches have been developed to address this problem. The present study explored the clinical potential of a novel photon-counting detector (PCD) CT system in reducing metal artifacts in head CT scans. In particular, we studied the recovery of an oral tumor region located under metal artifacts after correction. Three energy thresholds were used to group data into three bins (bin 1: low-energy, bin 2: middle-energy, and bin 3: high-energy) in the prototype PCD CT system. Three types of physical phantoms were scanned on the prototype PCD CT system. First, we assessed the accuracy of iodine quantification using iodine phantoms at varying concentrations. Second, we evaluated the performance of material decomposition (MD) and virtual monochromatic images (VMIs) using a multi-energy CT phantom. Third, we designed an ATOM phantom with metal insertions to verify the effect of the proposed metal artifact reduction. In particular, we placed an insertion-mimicking an iodine-enhanced oral tumor in the beam path of metallic objects. Normalized metal artifact reduction (NMAR) was performed for each energy bin image, followed by an image-based MD and VMI reconstruction. Image quality was analyzed quantitatively by contrast-to-noise ratio (CNR) measurements. The results of iodine quantification showed a good match between the true and measured iodine concentrations. Furthermore, as expected, the contrast between iodine and the surrounding material was higher in bin 1 image than in bin 3 image. On the other hand, the bin 3 image of the ATOM phantom showed fewer metal artifacts than the bin 1 image because of the higher photon energy. The result of quantitative assessment demonstrated that the 40-keV VMI (CNR: 20.6 ± 1.2) with NMAR and MD remarkably increased the contrast of the iodine-enhanced region compared with that of the conventional images (CNR: 10.4 ± 0.5) having 30 to 140 keV energy levels. The PCD-based multi-energy CT imaging has immense potential to maximize the contrast of the target tissue and reduce metal artifacts simultaneously. We believe that it would open the door to novel applications for the diagnosis and treatment of several diseases.

## Introduction

Dual-energy computed tomography (DECT) has provided new opportunities for X-ray CT. The major advantage of DECT is that it shows different attenuations of two materials using two different energy spectra, thereby allowing the separation of two materials that can hardly be distinguished by single energy CT [1, 2]. DECT reduces the use of contrast media [3, 4] as it improves the contrast among different tissues. Several applications of DECT have been reported since its introduction. For example, virtual monochromatic images (VMIs), synthesized from DECT data, are utilized to locate abdominal tumors (such as liver, pancreatic, and kidney tumors) [5–8], intracerebral hemorrhage [9], pulmonary embolism [10, 11], and vascular calcification [12, 13]. Moreover, VMI enables the detection and characterization of chemical compositions of materials that help in assessing specific disease processes [14]. However, the DECT approach suffers from certain limitations related to its mechanism of image acquisition. For instance, difference in the acquisition time of high- and low-energy datasets [2, 15] and acquiring images with substantial spectral overlap are major challenges.

Recently, photon-counting detector (PCD) CT scanners, which use an X-ray detector with improved energy-resolving power, have emerged as a diagnostic technique. This detector adopts a mechanism different from that used in conventional energy-integrating detectors; it uses a photon-counting technology. It counts the number of photons striking a detector and directly measures the photon energy. PCD-based multi-energy CT imaging is an innovative technology [16], which can considerably improve the current CT imaging through reduced radiation exposure [17], high spatial resolution [18–20], reduced beam-hardening artifact [21–23], and improved material decomposition (MD) [24, 25].

In the present study, we focused on the problem of artifacts caused by metallic dental prosthetics in head and neck CT that can degrade the image quality. Typically, dental fillings or implants generate severe streaks or shadings, hampering the accurate characterization of the type of tissue, which is critical when structures of diagnostic interest overlap with metal artifacts. As a result, it can be considerably challenging to the clinicians to determine the pathological condition [26–29]. Furthermore, metal artifacts could lead to inaccurate location of a tumor and wrong characterization of the surrounding tissues. Accurate delineation of tumors from the surrounding tissues is crucial in a radiotherapy plan for cancer treatment [30, 31].

To solve this problem, we used a prototype CT scanner based on a commercially available CT scanner installed with an in-house developed PCD system. Furthermore, we designed a dedicated phantom in which an insertion mimicking an iodine-enhanced oral tumor is placed between the two metallic dental implants. Despite iodine enhancement, the insertion is hardly detected by conventional single-energy CT owing to beam hardening or photon starvation of teeth, jawbone, and metallic dental implants. We evaluated the imaging performance and application of the PCD CT system for viewing the region of an oral tumor lying between highly dense metallic objects.

## Materials and methods

### Photon-counting detector CT scanner

**Fig 1:**
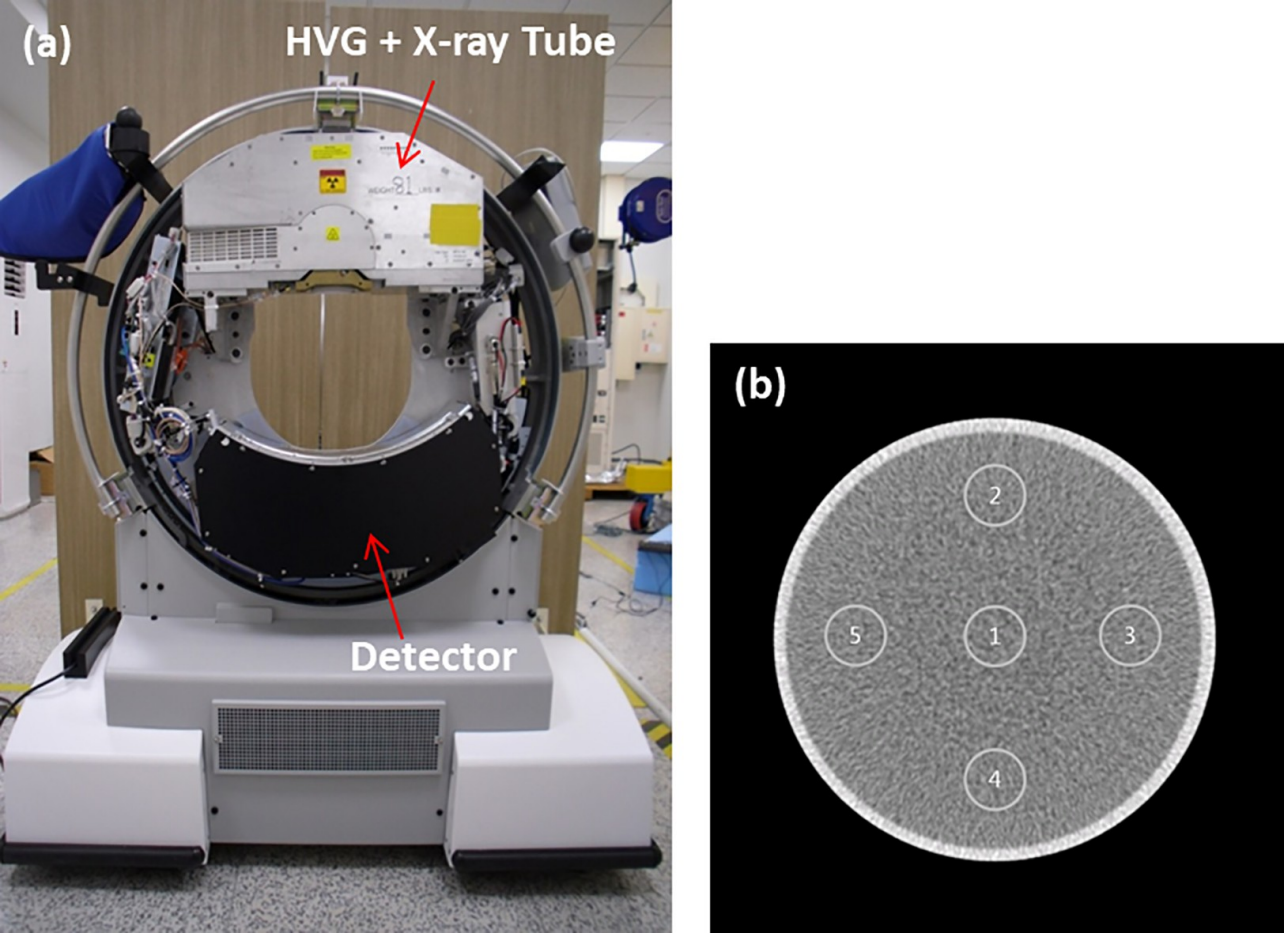
(a) Image of the prototype photon-counting CT system. (b) Illustration of ROIs used for uniformity measurement using the cylindrical water phantom with 150 mm diameter; uniformity value (the absolute value of [center (1) mean CT#-periphery (2–5) mean CT#]). 140 kVp, 1 mA, slice thickness: 0.625 mm, display window: W/L = 400/0 HU.

We developed a prototype PCD CT system based on a CT system available in the market (CereTom; Samsung-NeuroLogica, Danvers, USA) while using the original X-ray tube. The geometry of the scanner was as follows: the source to isocenter distance was 227.5 mm, and the source to detector distance was 408.6 mm. Hybrid multi-bin PCDs were integrated into this system, which operated on an application-specific integrated circuit (ASIC). The newly designed ASIC consisted of a 1.4-mm thick cadmium telluride (CdTe) sensor. The physical pitch of the detector pixel was 190 μm × 230 μm, and the anti-scatter collimator grid was placed between the pixels (every sixth pixel) to suppress the scattering on the plane. The effective size of the pixel along the z-axis at the isocenter was 0.128 mm. The detector assembly included 48 detector modules, with each detector module having an array size of 80 × 48 pixels. As a result, the detector assembly had 80 rows and 2,304 columns, allowing the collection of 80 simultaneous slices of data with each rotation of the gantry. The field of view (FOV) of the scanner at the isocenter was 250 mm in diameter (Fig 1A).

We investigated the characteristics of our CdTe detector, such as energy resolution, count rate (detector efficiency), and detector stability. First, we measured the energy resolution using radioactive isotopes of Co-57 with photon energy of 122.06 keV. Energy spectra were obtained using a threshold scan of radioactive isotopes. We swept the energy threshold and obtained all counts above the threshold energy. Energy spectra were extracted by differentiation. Photopeaks were obtained by fitting a Gaussian function in the spectra. Next, energy resolution was calculated from the full width at half maximum (FWHM) of the photopeak in the spectrum divided by its energy. Second, we conducted experiments to determine the count rate. Scan parameters were obtained under a single-channel image (energy range above 30 keV) with varying tube currents (25, 15, 25, 55, 73, and 91 mA) at 120 kVp tube voltage, and 1 s data acquisition time. The data were fitted to a non-paralyzable counter model to characterize the time response. The true (input) count rate n is given by n = m/(1−mτ) [32], where m is the measured count rate and τ is the dead time. The measured count rate was obtained by averaging the 25 detector pixels. Finally, the behavior of the detector response for a certain period was determined by measuring the response for 20 s with a –900-V bias voltage at the same scan parameters as that used for the analysis of the count rate.

### Image acquisition

Our PCD CT system generated three-energy bins with an arbitrary keV setting. Energy calibration using the polyenergetic spectrum was performed to set the thresholds of all pixels. We used two additional filters, gadolinium and tungsten having different K-edge energies, 50.2 keV and 69.5 keV, respectively. From the measured X-ray spectra, threshold values corresponding to the K-edge energies of individual pixels were acquired using a linear fitting. We applied 30 keV, 50 keV, and 65 keV as thresholds for energy bin 1, bin 2, and bin 3, respectively. Images were acquired in the 5 × 6 binning mode. The slice thickness at the isocenter was 0.640 mm, and the number of slices per scan was 16. Axial scans were obtained under conditions of 140 kVp voltage, 4 mA current, gantry rotation time of 2 s, and 1,440 projections per rotation.

### Phantom

**Fig 2:**
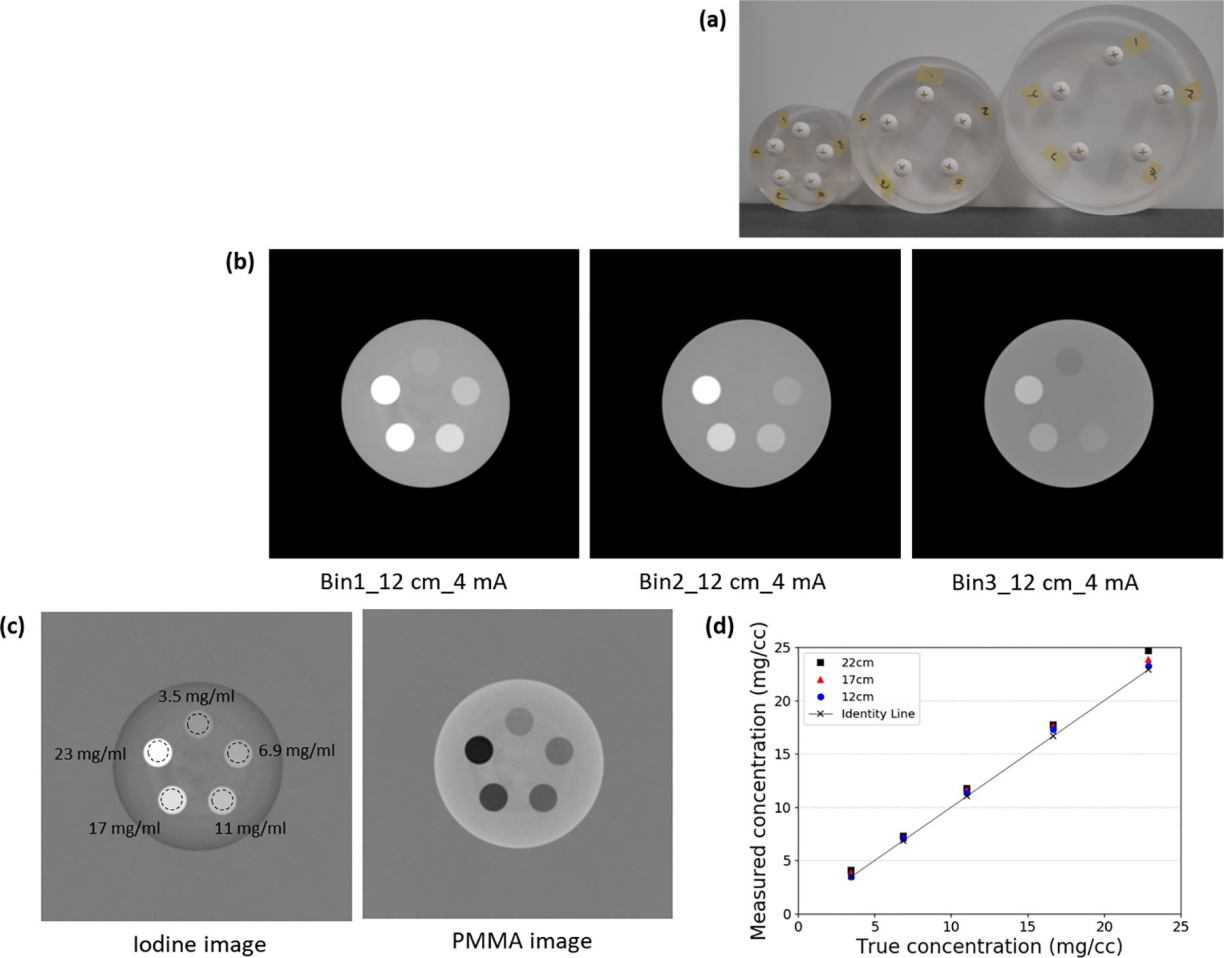
(a) Three different cylindrical phantoms (diameter: 12, 17, and 22 cm) for iodine quantification accuracy. (b) Low-energy threshold (bin 1: 30–50 keV), middle-energy threshold (bin 2: 50–65 keV), and high-energy threshold (bin 3: 65–140 keV) images of the 12 cm phantom. (c) Iodine images and PMMA images. (d) Comparison of the measured iodine concentration with the true concentration.

**Fig 3:**
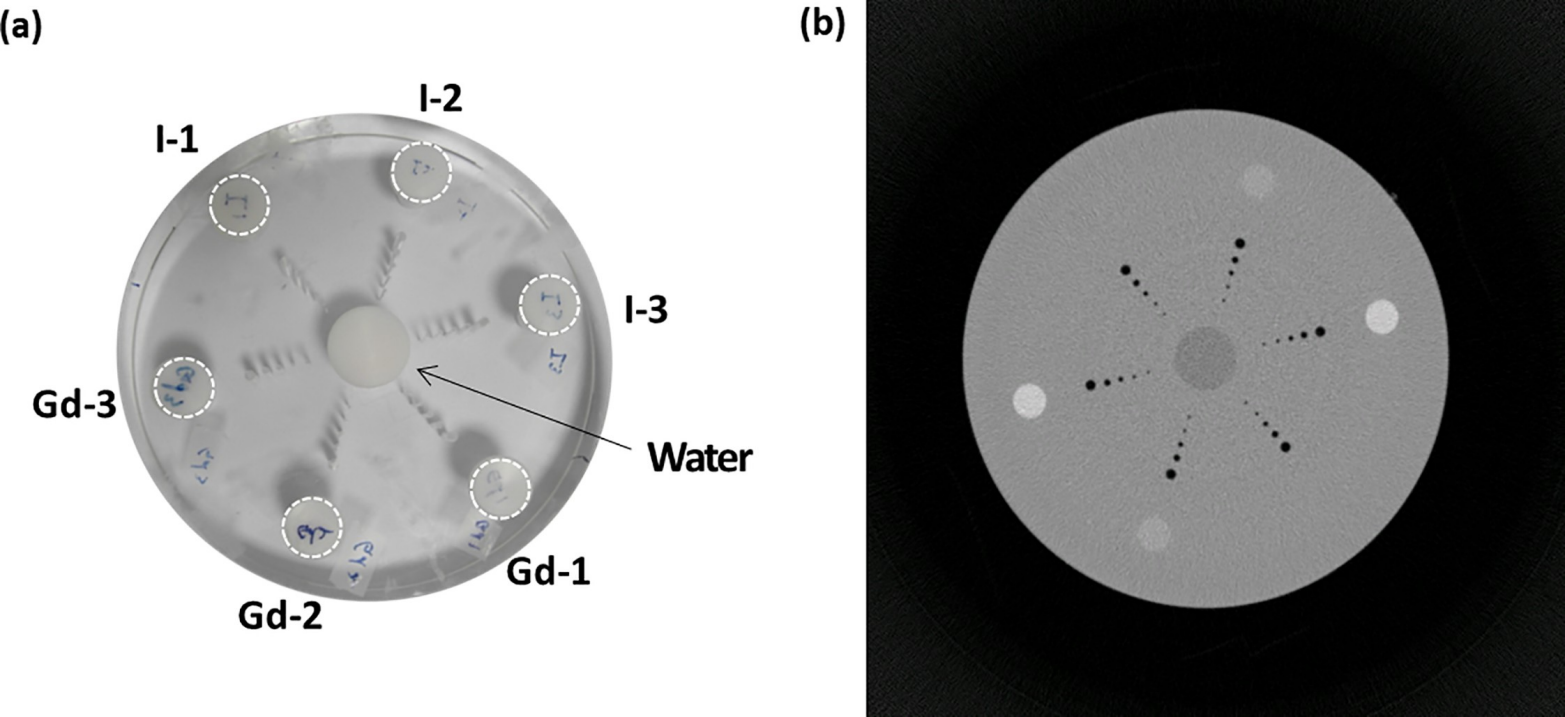
(a) Cylindrical PMMA phantom with 150 mm diameter and (b) transaxial image at a single-channel image for the 30–140 keV energy level. The phantom was specifically designed for basis material calibration and contained drilled holes filled with iodine (I) and gadolinium (Gd) at several concentrations: iodine (I-1: 3.7 mg/mL, I-2: 10.1 mg/mL, I-3: 18.9 mg/mL) and gadolinium (Gd-1: 3.0 mg/mL, Gd-2: 7.5 mg/mL, Gd-3: 13.8 mg/mL).

**Fig 4:**
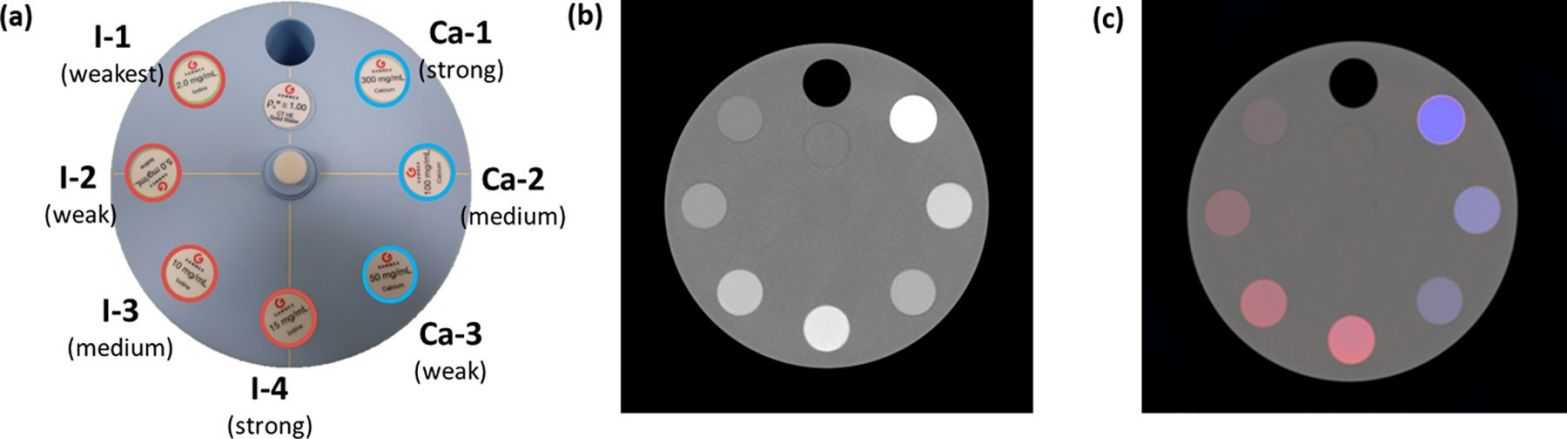
(a) Gammex phantom with iodine and calcium at various concentrations: Iodine (I-1: 2.0 mg/mL, I-2: 5.0. mg/mL, I-3: 10 mg/mL, I-4: 15 mg/mL) and calcium (Ca-1: 300 mg/mL, Ca-2: 100 mg/mL, Ca-3: 50 mg/mL). (b) The single channel image for the 30 to 140 keV energy level (display window: W/L = 1000/0 HU): Iodine (I-1: 51 HU, I-2: 128 HU, I-3: 268 HU, I-4: 402 HU) and calcium (Ca-1: 899 HU, Ca-2: 333 HU, Ca-3: 195 HU). Calcium and iodine are difficult to distinguish. (c) An overlaid image that clearly separates iodine and calcium using images of the three energy levels (bin 1, bin 2, and bin 3) (pink: iodine, blue: calcium).

**Fig 5:**
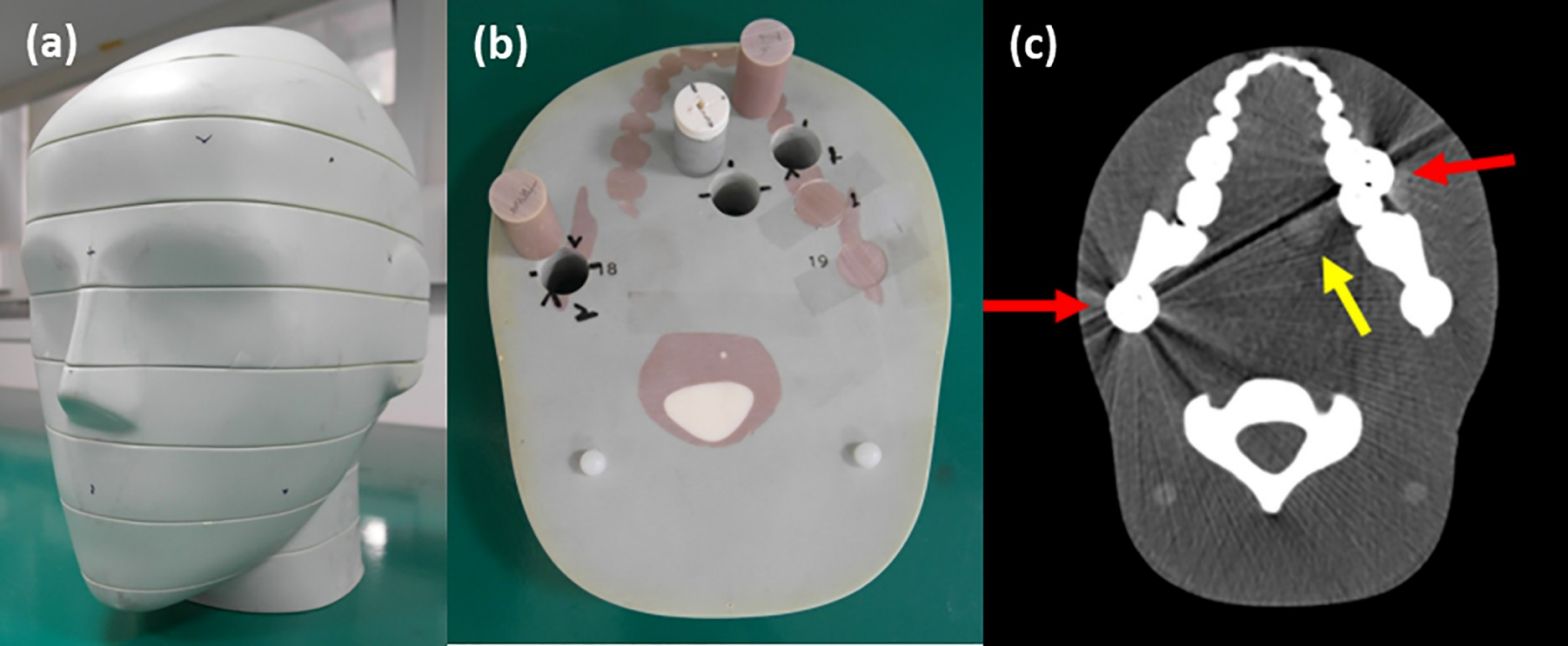
(a) Adult male ATOM head phantom (ATOM phantom) that artificially generates metal artifacts and targets iodine-enhanced oral tumor. (b) Two metal implants and iodine were inserted into the ATOM phantom. (c) Image for the 30 to 140 keV energy level. We placed two metallic implants, one on the left, and the other on the right region of the lower jawbone (red arrow); iodine-enhanced tissue (yellow arrow) is located between them. Display window: W/L = 600/150 HU.

We measured uniformity, one of the fundamental image quality metrics, using a cylindrical water phantom with a diameter of 150 mm. As shown in Fig 1B, CT numbers were measured on regions of interest (ROIs) at the center and four peripheries to assess uniformity. To evaluate the accuracy of iodine quantification, we built cylindrical phantoms with five holes (20 mm diameter) to be filled with iodine solutions of varying concentrations. The cylindrical phantoms were made of polymethyl methacrylate (PMMA) and had diameters of 12, 17, and 22 cm (Fig 2). Iodinated contrast medium (Iomeron 350 mg/mL; Bracco, Milan, Italy) was diluted with water to produce iodine concentrations of 3.5, 6, 9, 11, 17, and 23 mg/mL. Next, the holes of the phantoms were filled with diluted iodine. We first applied MD and measured the density of iodine contrast from iodine-decomposed images of cylindrical phantoms. The decomposed iodine and PMMA images of the cylindrical phantom are shown in Fig 2C. To decompose materials more accurately, we used another phantom specifically designed for basis material calibration, which is described in Fig 3. The true concentration of iodine in the calibration phantom was 18.9. mg/mL, based on which the values of the images could be converted relatively. In other words, a converted value of 1 indicated full contrast of 18.9 mg/mL. The largest possible circular ROI without touching the container was drawn to measure the concentration of iodine in each of the five holes. This process was applied to the images of each phantom size. The measured iodine concentration was compared to the ground truth, namely the real known concentration in each hole, and errors were calculated. A linear fit was performed, and the coefficient of determination (R^2^) was reported. As shown in Fig 4, we used a multi-energy CT phantom (model 1472 Gammex) to evaluate the performance of MD and VMI. Fig 4a shows four solid insertions representing four concentrations of iodine (I-1 to I-4) and three insertions of calcium (Ca-1 to Ca-3). PMMA and Gammex phantoms were scanned in the axial scan mode with 140 kVp tube voltage, 4 mA tube current, and a speed of 2 s per rotation. We inserted two titanium implants in the adult male ATOM head phantom (model ATOM 701-HN; CIRS Inc., VA) (Fig 5), one on the left and the other on the right region of the lower jawbone (Fig 5C, red arrows). Furthermore, we placed an iodine-enhanced tissue (Fig 5C, yellow arrow) between the implants to simulate a challenging situation for the overlap of the tissue and streak artifacts. The Hounsfield unit (HU) values of inserted iodine and the surrounding tissues were 75 and 57, respectively.

### Metal artifact reduction for multi-energy CT

**Fig 6:**
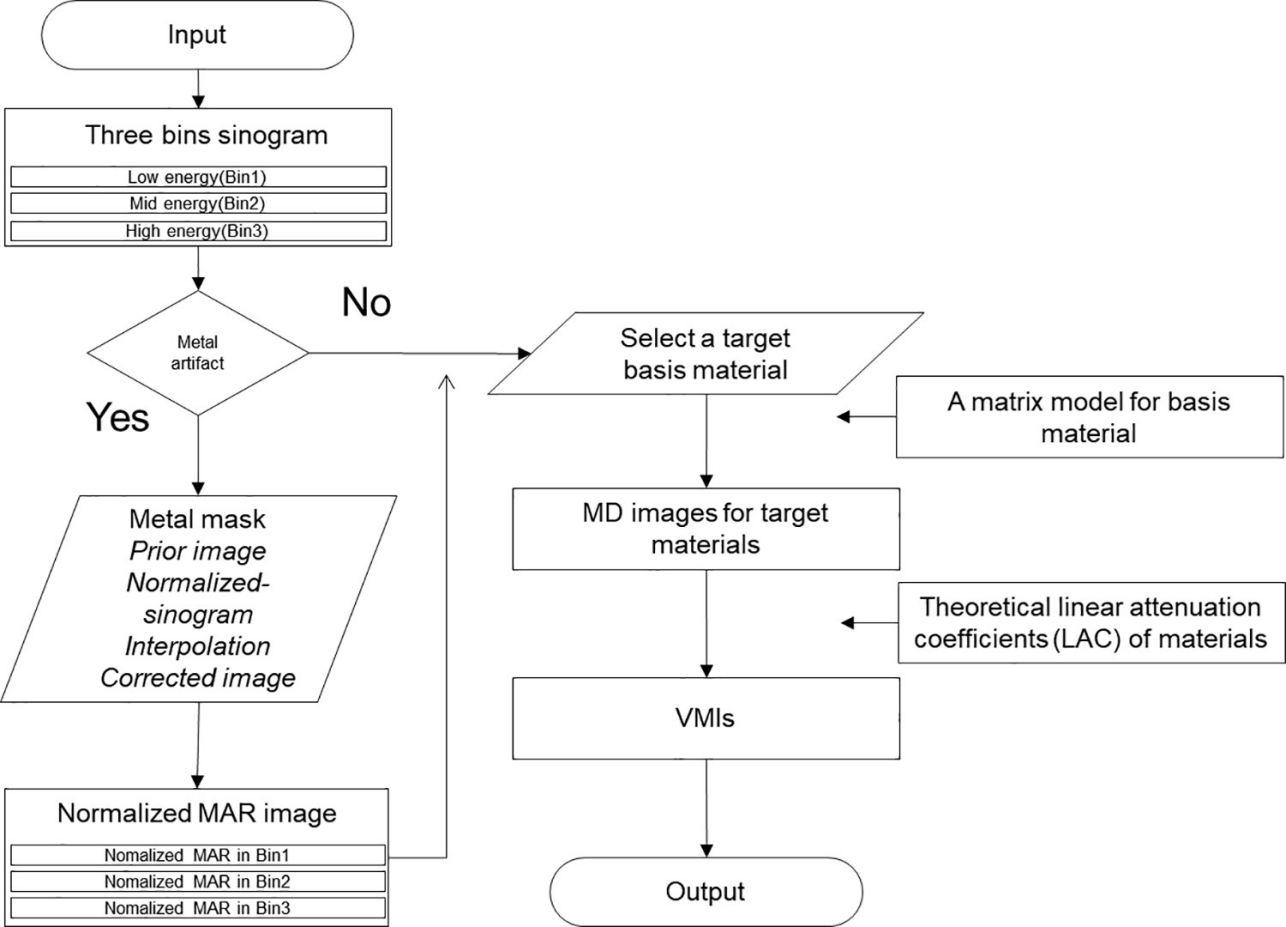
Scheme of metal artifact reduction and tissue characterization image for multi-energy CT. The NMAR method and MD are applied to the bin 1, bin 2, and bin 3 reconstructed images of different energy levels and created VMIs.

The normalized metal artifact reduction (NMAR) method [33] was applied before performing MD and VMI reconstruction. Metallic objects were identified in the image domain through thresholding, and they were forward projected to find the corresponding metal trace in the projection domain. Subsequently, a prior image was obtained by segmentation using multiple thresholding of the bone, soft tissue, and air regions. A miss-segmentation between air and the bone (such as the boundary of the tooth) can lead to new streaks of artifacts. To prevent such artifacts, we applied a morphological operation with a circular kernel on multiple segmentations. The modified NMAR was applied to each of the three multi-energy images for comparison with an integrated image of 30 to 140 keV energy level. Conventional MD based on three materials (water, iodine, and bone) was utilized, following which VMIs were created by the weighted sum of three material maps. The overall scheme of the proposed method is shown in Fig 6.

### Algorithms for MD and VMI

The aim of MD is to find *b_m_*(*x*) for each image pixel x based on the formula below:

$$\mu^{i}(x)=\sum_{m=1}^{M}b_{m}(x)\mu_{m}^{i},i=1,2,3$$ (1)

where *μ^i^*(*x*) is the attenuation coefficient of the reconstructed image for the *i*-th energy bin, M is the number of the basis material, *b_m_*(*x*) is the mass fraction of the basis material lying in the range of [0,1], and $\mu_{m}^{i}$ is the known attenuation coefficient of each basis material at the *i*-th energy bin. In the case of three materials, this can be solved by a matrix inversion from the formula:

$$\left[\begin{array}{c} \mu^{1}(x)\\ \mu^{2}(x)\\ \mu^{3}(x) \end{array}\right]=\left[\begin{array}{ccc} \mu_{1}^{1} & \mu_{2}^{1} & \mu_{3}^{1}\\ \mu_{1}^{2} & \mu_{2}^{2} & \mu_{3}^{2}\\ \mu_{1}^{3} & \mu_{2}^{3} & \mu_{3}^{3} \end{array}\right]\;\left[\begin{array}{c} b_{1}(x)\\ b_{2}(x)\\ b_{3}(x) \end{array}\right]$$ (2)

**Fig 7:**
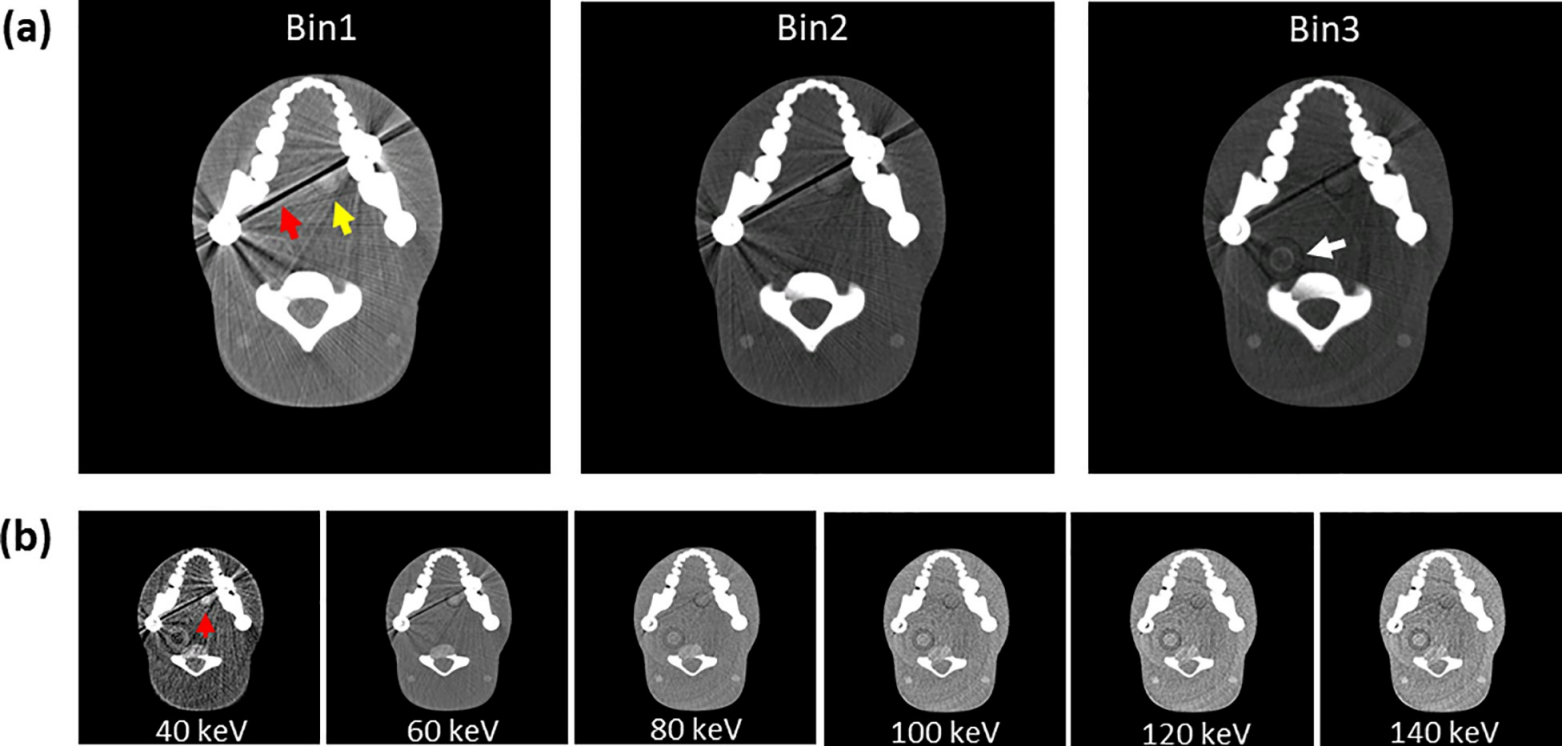
Multi-energy bin 1, bin 2, and bin 3 images acquired from the PCD CT system. VMI was created using three acquired images. (a) The higher the energy from bin 1 to bin 3, the smaller the metal artifact (red arrow). Compared with bin 2 and bin 3, bin 1 (yellow arrow) shows enhanced iodine in the oral tumor portion. (b) VMI from 40 keV to 140 keV created from three energy bin images reconstructed from an iodine-enhanced ATOM phantom with a left oral tumor (red arrow). VMIs of the same scan/slice are shown at 40, 60, 80, 100, 120, and 140 keV. Display window: W/L = 600/150 HU.

The VMI is reconstructed using the formula below.

$$\mu (x,E)=\sum_{m=1}^{M}b_{m}(x)\mu_{\rho ,m}(E)$$ (3)

where *M* is the number of basis material, *b_m_*(*x*) is the mass fraction of the basis material (the result of MD), and *μ_ρ,m_*(*E*) is the mass attenuation coefficient of the *m*-th material at energy *E*. The result of VMI using this formula is shown in Fig 7B.

### Evaluation of image quality

**Fig 8:**
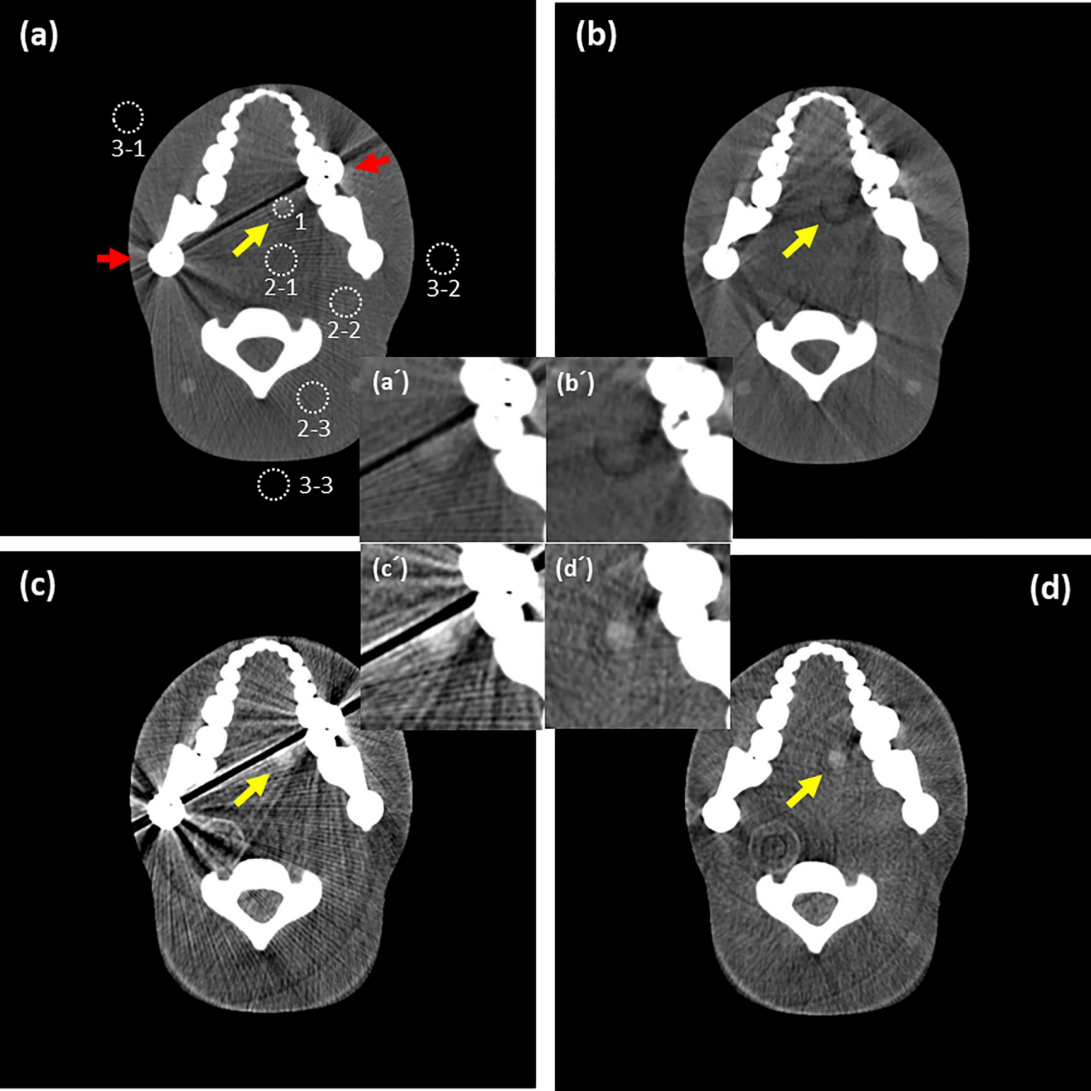
(a) Imaging of adult male ATOM phantom acquired at an energy level of 30 to 140 keV. White dotted lines outline the ROIs placed for objective image analysis: ROI1, area of iodine-enhanced tissue; ROI2s, normal tissue (area where metal artifact is less affected), and ROI3s surrounding air for measuring background noise. (b) Image with NMAR method applied to image A. (c) Virtual monochromatic image of 40 keV with MD applied to bin 1, bin 2, and bin 3 without NMAR. (d) Iodine-enhanced virtual monochromatic image (yellow arrow) at 40 keV, which applied NMAR and MD to bin 1, bin 2, and bin 3. The imaging shows how metal artifacts were removed and iodine was enhanced. (a´), (b´), (c´), and (d´) are magnified images of (a), (b), (c), and (d) focusing on the iodine-enhanced tissue. Display window: W/L = 600/150 HU.

Seven ROIs were selected in the image of the ATOM phantom (Fig 8), including an area where metal artifacts were most severe. The mean CT number (in HU) and standard deviation (SD, i.e., image noise) were measured in the circular ROIs: ROI_1_, area of iodine-enhanced tissue; ROI_2s_, normal tissue (area where metal artifact is less affected), and ROI_3s_ surrounding air for measuring background noise. Contrast-to-noise ratios (CNRs) were calculated using the following formula: CNR = (ROI_1_–ROI_2s_)/ROI_3s_ (or background noise) [34, 35].

## Results

**Fig 9:**
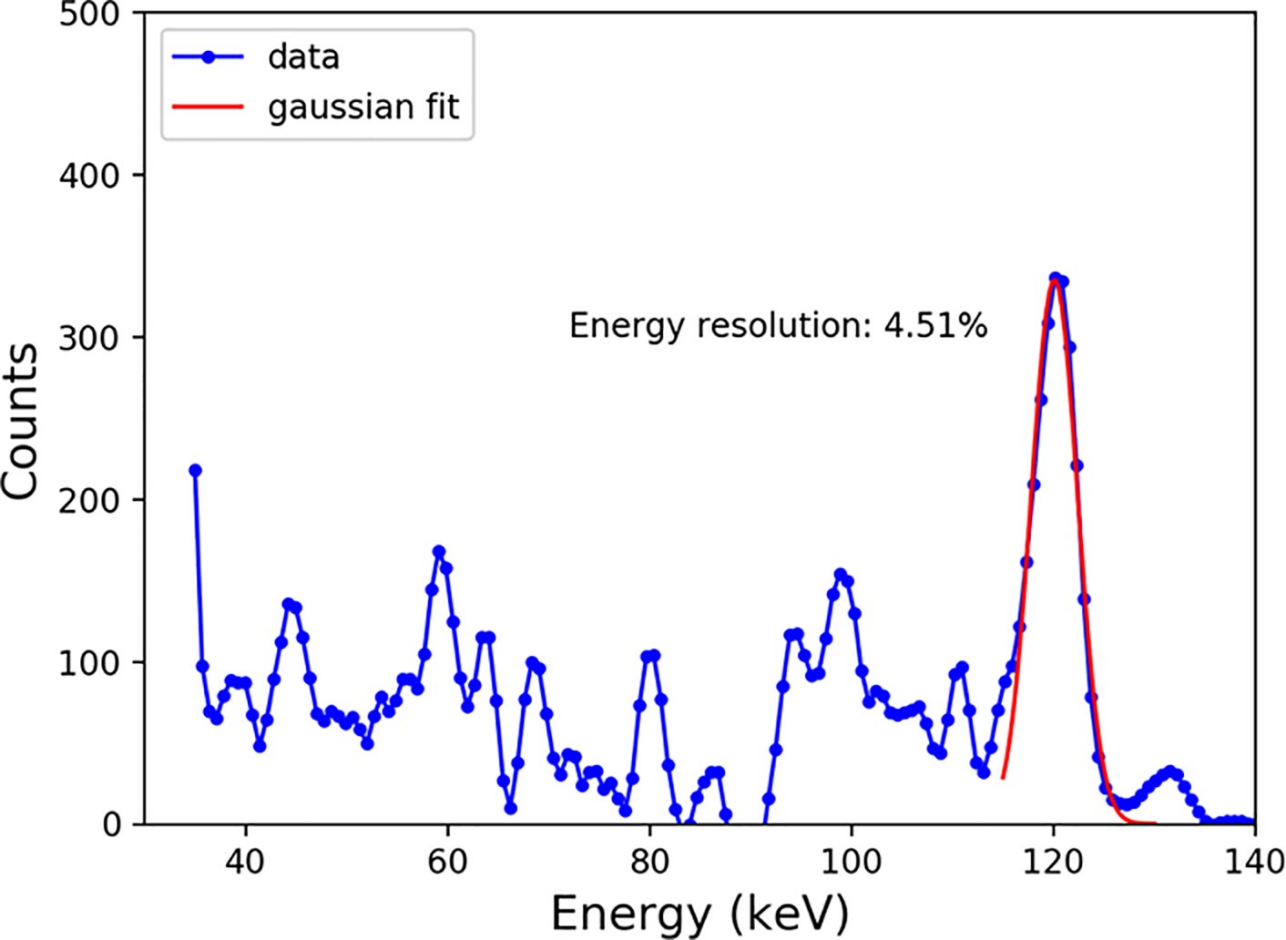
The energy spectrum of the CdTe detector with FWHM of 4.51% at 122.06 keV.

**Fig 10:**
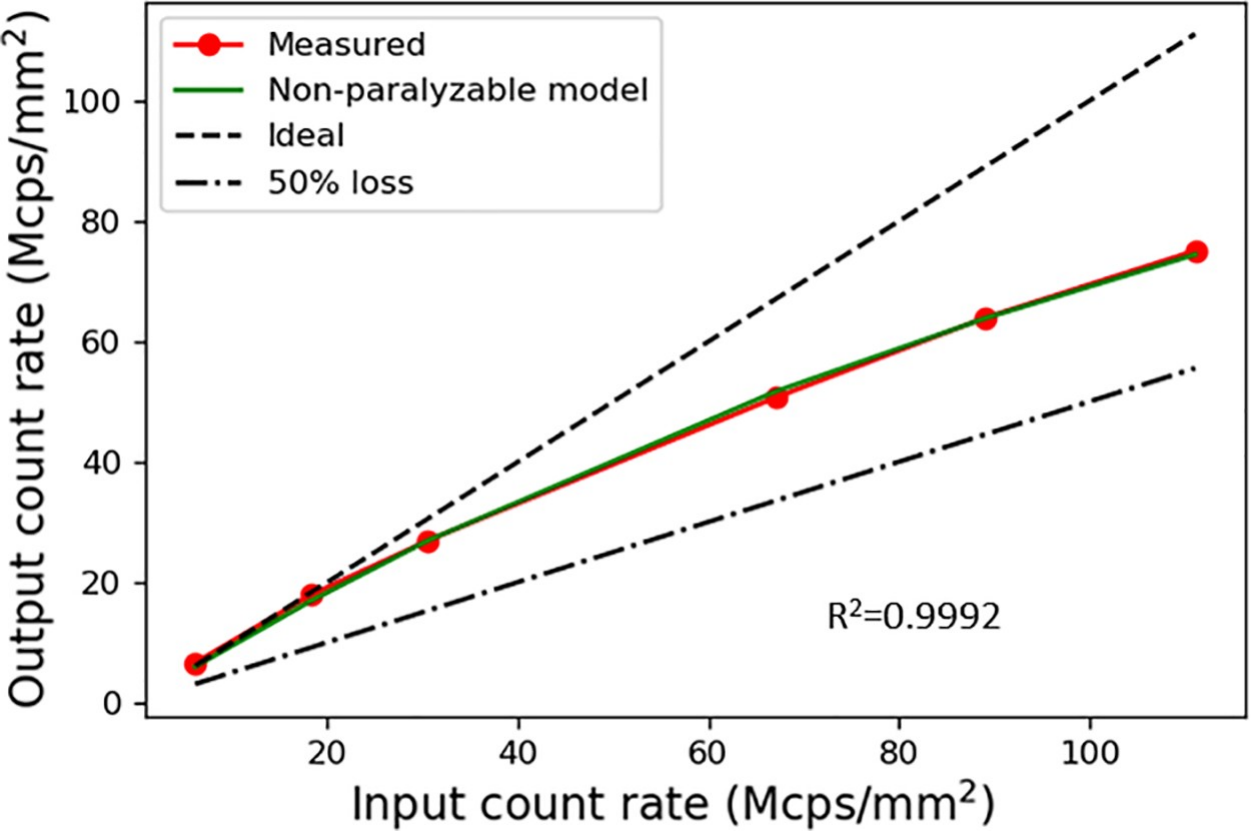
Count rate performance of the CdTe detector. The output count rates were obtained by averaging the 25 detector pixels. The count rate was recorded as a function of input count rate (5, 15, 25, 55, 73, and 91 mA) for 120 kVp tube voltage, and 1 s data acquisition time.

**Fig 11:**
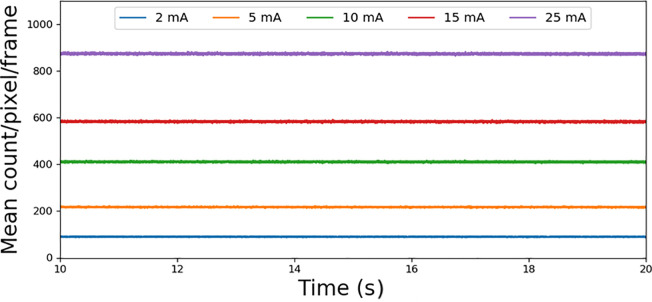
Stability of mean pixel (25 pixels) counts per frame in 20 s for varying tube currents at 120 kVp tube voltage. Mean pixel counts and SD were 90 ± 1.0 (2 mA), 217 ± 1.4 (5 mA), 411 ± 1.9 (10 mA), 583 ± 2.1 (15 mA), and 874 ± 2.5(25 mA), respectively.

The estimated energy resolution reached 4.51% at 122.06 keV using the 1.4-mm thick CdTe detector, as depicted in Fig 9. Fig 10 shows the averaged output count rate of 25 detector pixels as a function of input count rates. It implies an average non-paralyzable dead time (τn) of 101.42 ns and count rate per detector area of 112.81 Mcps/mm^2^ (at 50% loss). The mean counts of pixels per frame as a function of time were calculated as a measure of the stability, and the result is shown in Fig 11. It appears that the mean count was considerably stable in the 20-s time window. Its standard deviation was less than 1.1% for several tube current conditions. Further investigation is necessary to understand the meaning of the measured stability for several CT protocols and applications.

### Accuracy and uniformity of iodine quantification

The iodine phantoms were scanned using a prototype PCD system. Fig 2B shows images at each energy bin (bin 1: 30–50 keV, bin 2: 50–65 keV, and bin 3: 65–140 keV) of the 12 cm phantom. As expected, the image at lower energy provides an increased contrast between iodine and its surrounding materials. This is because the k-edge of iodine (33.2 keV) was closer to bin 1 than to bin 2 or bin 3. As a result, the attenuation of iodine-containing substances was substantially higher than in the background at bin 1.

Fig 2C shows the material separated iodine and PMMA images. The overall trend shows a good match with the ground truth. As shown in Fig 2D, a linear relationship was observed between the measured and true iodine concentrations for the PCD CT system (R^2^> 0.99). The errors between the measured and true iodine concentrations ranged from 0.4 to 1.8 mg/mL, which corresponded to a maximum of 7.5% error. This was comparable to the results of a previous study [36]. The uniformity in the water phantom was acceptable; more specifically, the maximum difference in the mean HU values on five ROIs was 4.3 HU, lying within the clinically acceptable range (±5 HU), as shown in Fig 1B.

### Material decomposition and VMI

We used the Gammex phantom-based experiment to evaluate the performance of our PCD system for MD. Fig 4B shows a single channel image (30–140 keV) in which some solid insertions are not clearly distinguished by HU values in the window setting W/L (1000/0). In particular, the HU values for I-3, Ca-2, I-2, and Ca-3 were considerably similar. In contrast, Fig 4C shows a clear separation of the solid insertions of iodine and calcium using an overlaid image based on the results of MD.

We conducted a further experiment using the ATOM phantom to evaluate the overall multi-energy performance. Reconstructed images at three different energy bins, shown in Fig 7A, demonstrate that the strength of metal artifacts (red arrow) decreased as the mean energy increased from bin 1 to bin 3. We still have few remaining rings (white arrow) in the bin 3 image owing to imperfect calibration. The results of VMI approach are demonstrated in Fig 7B for energies ranging from 40 keV to 140 keV with a 20-keV interval. The tradeoff between the contrast of the iodine-enhanced insertion and the severity of metal artifacts is well evident. While the contrast is maximized in the 40-keV image, it suffers from severe metal artifacts that partially overlap with the lesion. On the contrary, while metal artifacts tend to mitigate as the energy increases, the tumor-mimicking lesion is hardly detected in the images above 100 keV. This is where the idea of applying NMAR comes into play. We applied NMAR to the three bin images and generated VMIs to achieve both the maximum contrast and reduced metal artifact simultaneously.

**Fig 12:**
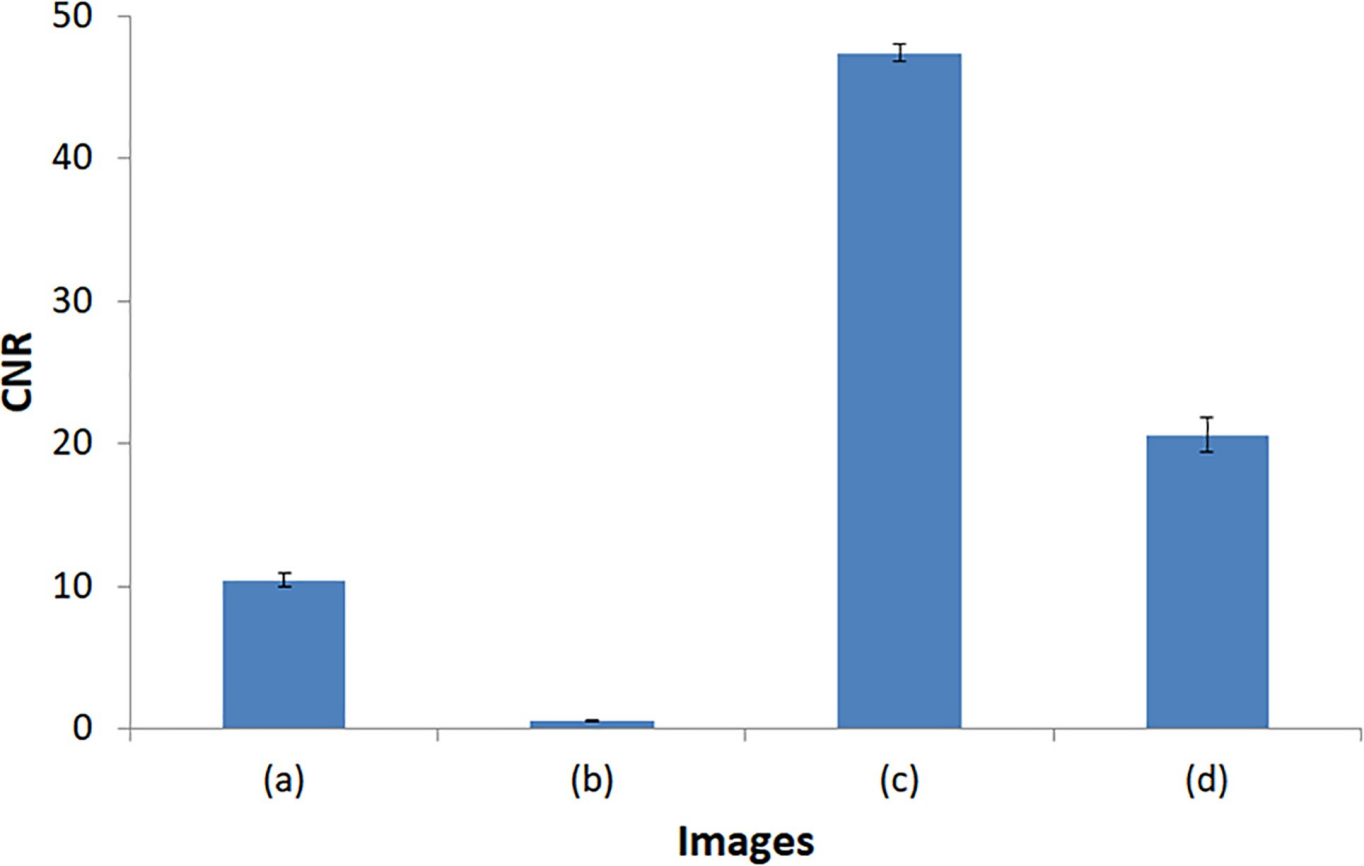
Contrast-to-noise ratio (mean ± standard deviation) for quantitative image quality of the multi-energy CT imaging in Fig 8.

For comparison with the conventional approach, we demonstrated four images in Fig 8. Fig 8A is an image of the ATOM phantom acquired from 30 to 140 keV, and (b) is the result of NMAR applied to (a). Fig 8C shows the VMIs of 40 keV generated due to MD using bin 1, bin 2, and bin 3 without applying the NMAR method. Applying NMAR to each energy bin first generates the VMI at 40 keV in (d), which demonstrates the effect of the proposed method. Unlike the conventional approach, metal artifacts are removed and the lesion is well visualized despite certain residual artifacts. The result is promising as it can be utilized to view lesions when highly dense materials are present in the field of view (FOV). Table 1 and Fig 12 show the detailed mean ± SD and CNR of each ROI in Fig 8.

**Table 1:** Detailed values (HU) of mean ± SD and CNR at each ROI. These are used for quantitative evaluation of image quality of the multi-energy CT imaging shown in Fig 8.

Images	ROI_1_	ROI_2-1_	ROI_2-2_	ROI_2-3_	ROI_3-1_	ROI_3-2_	ROI_3-3_	CNR1	CNR2	CNR3
(a)	103.1 ± 19.8	24.8 ± 10.0	21.0 ± 15.8	27.7 ± 9.7	–997.0 ± 8.4	–999.9 ± 6.5	–1008.7 ± 7.7	10.4	10.9	10.0
(b)	29.1 ± 11.3	25.8 ± 13.7	25.5 ± 17.5	25.3 ± 7.0	–998.1 ± 6.8	–1004.7 ± 5.7	–1002.8 ± 5.9	0.5	0.6	0.6
(c)	360.8 ± 65.7	33.9 ± 40.2	41.5 ± 41.4	35.2 ± 36.7	–1000 ± 7.6	–1000 ± 6.1	–1000 ± 6.8	47.9	46.8	47.7
(d)	178.0 ± 25.9	40.7 ± 27.2	43.4 ± 22.8	28.1 ± 21.5	–1000 ± 7.6	–1000 ± 6.1	–1000 ± 6.8	20.1	19.7	22.0

## Discussion

Metal prosthetics inserted into the human body can cause artifacts that severely distort or compromise the quality of CT images. To address the problem, several approaches have been proposed, such as algorithms reducing metal artifacts, reconstruction using thin slices, and scanning with a high voltage. However, each of these show limitations [37]. Recently, dual-energy CT has been used to solve the problem of metal artifacts. One can create VMIs at an arbitrary energy level ranging from 40 to 140 keV after collecting the DECT data. Monochromatic images at higher energy (minimum 100 keV) develop less metal artifacts owing to high photon energies. Moreover, they outperform conventional polychromatic images or monochromatic images at lower energy [27–29]. However, current DECT suffers from non-ideal workflow, limitations in energy separation, etc. Our PCD CT system can generate three energy bins with different keV settings. To equalize the ratio of photons at each energy bin, we applied thresholds of 30 keV, 50 keV, and 65 keV to energy bin 1, bin 2, and bin 3, respectively.

In the present study, we explored the potential of spectral CT imaging based on a PCD CT system for visualizing iodine-enhanced oral tumors when metallic objects are present in head and neck CT. Customized phantoms were scanned in a prototype PCD CT system with three energy thresholds. Afterward, metal artifact reduction, MD, and virtual monochromatic imaging are performed. We first removed metal artifacts by applying the NMAR methods to bin 1, bin 2, and bin 3. Next, we created VMIs using materially decomposed imaging.

Fig 7 shows the behavior of metal artifacts depending on the energy level. Both bin images (a) and VMI (b) show that metal artifacts decrease as the energy level increases; this is opposite to the behavior of soft tissue contrast. In summation, images at lower energy have higher contrast and can be hardly distinguished from its background in images at higher energy in iodine-enhanced insertion. We could maximize the visualization by combining VMI and reduction in metal artifact, which is a remarkable advantage over the conventional approach. Whereas the images of the conventional energy-integrating CT system suffer from metal artifacts or poor contrast (CNR: 10.4 ± 0.5 and 0.6 ± 0.0) as shown in Fig 8A and 8B, respectively, images of the novel PCD CT system show an outstanding improvement in CNR (47.5 ± 0.6, 20.6 ± 1.2), as shown in Fig 8C and 8D. Despite the metal artifact correction, the contrast of the lesion is extremely poor, making it difficult to identify its location as shown in Fig 8B. The reconstructed VMI (Fig 8C) at 40 keV without the NMAR method shows that the iodine enhancement is maximized in the tumor-mimicking area; however, the metal artifacts still exist. Fig 8D demonstrates the benefit of the proposed method. VMI at 40 keV reconstructed after applying the NMAR methods showed considerably reduced metal artifacts and maximized iodine enhancement in the tumor-mimicking area (yellow arrow in Fig 8D), which is not the case in the images obtained using the conventional method.

We can provide new opportunities for several CT-based treatments, such as cancer treatment, by selecting the lesion-specific energy level and adopting metal artifact reduction technology. Application of several de-noising strategies and improving the calibration are ongoing processes and warrant further research.

Our custom-developed PCD consisted of multiple hybrid chips that combined a CdTe chip with an ASIC. However, despite its disruptive concept, the resulting images have certain visible and remaining artifacts owing to certain limitations of PCD, such as charge sharing, pulse pileup, and fluorescence. We believe that improvements in the hardware and more sophisticated calibration could overcome these limitations.

**Fig 13:**
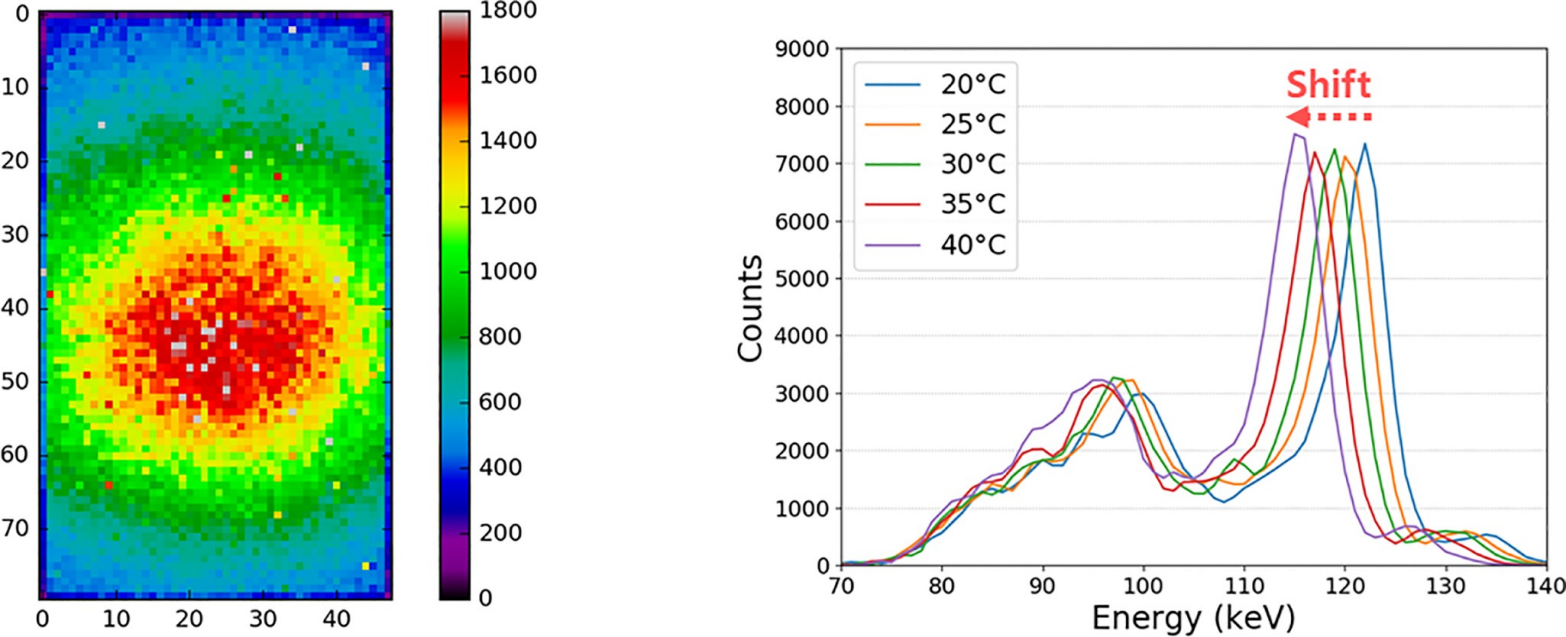
(a) Hybrid chip image using Co-57 (3.7 MBq) source. (b) Energy peak according to temperature. As the temperature rises, it shifts to the left.

One factor to be considered is the temperature of the PCD, which could distort the energy spectrum. It is well known that CdTe and ASIC are sensitive to temperature. When each hybrid chip has a different temperature, distortions and differences in the measured signals among the hybrid chips can easily occur. As a result, the rings and band artifacts remain in the final imaging even after calibrations and post-processing. To determine the effect of temperature change, we conducted an experiment as shown in Fig 13. We observed that the energy peak of the radioisotope, Co-57 (122.2 keV), shifted as the temperature was increased from 20°C to 40°C. This implied that PCD without a sophisticated cooling system could generate different detector responses even under the same scan conditions. Calibration tables generated at a different time might not work owing to the lack of reproducibility of the signals. Evaluating the impact of temperature on PCD could be an interesting research topic.

The present study has several limitations. First, the precise composition of the two dental implants in the ATOM phantom was not known, except that its basis was titanium. Several clinically relevant materials need to be assessed because the severity of beam hardening and photon starvation are strongly related to the type and size of the metal [38, 39].

**Fig 14:**
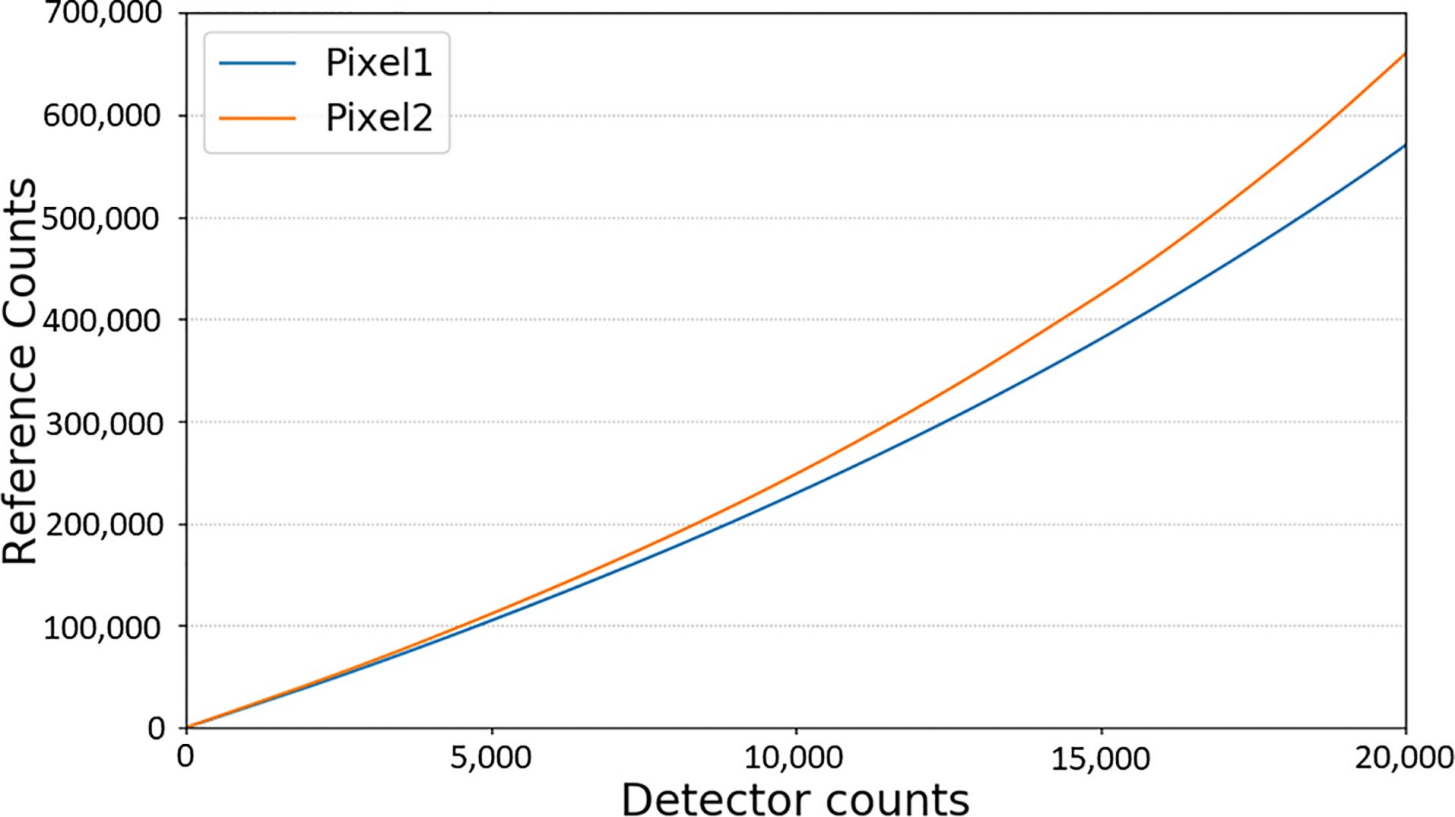
Detector–response curves of two different detector pixels. This non-linear behavior requires a sophisticated non-linearity correction, unlike the conventional energy-integrating detector.

Second, as is well known, the detector counts registered in the PCD CT system are not linearly correlated to the actual X-ray counts incident to the detector pixels (due to, e.g., pulse pileup). Therefore, to reconstruct CT images from this type of data, we need to correct this nonlinearity of the PCD data. For the pre-processing of each energy bin, we collected detector count data of several X-ray doses (different mAs). In addition to the detector counts, we collected one common reference count for each dose, known to be linearly correlated to X-ray doses. Fig 14 shows the detector–response curves used for this nonlinearity correction for two selected detector pixels. Unfortunately, “air correction” alone did not result in “artifact-free” reconstructed images. To remedy this situation, we used water phantom data to further reduce ring artifacts. Although the image quality improved, the pre-processing was an ongoing work. We can see the remaining ring and band artifacts at the 7 o’clock position in the middle of the bin 3 imaging in Fig 7A. As VMIs are reconstructed using bin 1, bin 2, and bin 3, inherited ring and band artifacts are pronounced in the same position in Fig 7B imaging. As mentioned above, we expect to stabilize the image quality by improving the hardware and using more sophisticated calibration. In particular, we look forward to evaluating the basic image quality after controlling the temperature of each hybrid chip.

Third, the performance of the proposed method may vary depending on the tissue of interest and the types of metal present in the FOV. As Cha [29] pointed out, the performance of VMI in combination with MAR is directly related to the energy selected to reconstruct monochromatic images. We can optimize the thresholds defining the energy bins depending on the applications, more specifically, on the target body structures. Furthermore, application of adaptive selection of thresholds is an interesting research topic for PCD systems. We will conduct more studies in this regard. Moreover, comparison with commercially available DECT systems is a potential future work.

## Conclusions

We developed a PCD CT system by integrating PCD to the commercially available CereTom and evaluated its potential benefits in head and neck CT. The proposed method in the PCD system showed great potential to provide enhanced iodine imaging of the simulated oral tumor with substantially reduced metal artifacts. We believe that PCD-based multi-energy CT imaging will open the door to new opportunities in the diagnosis and treatment of cancer in the head and neck.
